# New‐Onset Psychosis in a Person with Parkinson's Disease after “Horny Goat Weed” Use

**DOI:** 10.1002/mdc3.70350

**Published:** 2025-09-08

**Authors:** Juan R. Deliz, Kasra Manoocheri, Danielle N. Larson, Danny Bega

**Affiliations:** ^1^ Department of Neurology Parkinson's Disease and Movement Disorders Center, Northwestern University Chicago Illinois USA; ^2^ Department of Psychiatry Northwestern University Chicago Illinois USA

**Keywords:** Parkinson disease psychosis, epimedium toxicity

Neuropsychiatric symptoms (NPS) are common in Parkinson's disease (PD) and associated with disease progression and cognitive decline.^1^, ^2^ PD psychosis (PDP) encompasses a spectrum of psychotic phenomena and can manifest early in PD.^3^ PDP results from widespread structural, functional, and neurotransmitter changes, and may be exacerbated by dopaminergic or other central nervous system (CNS)‐acting compounds.^3^ Over‐the‐counter supplements, such as epimedium—the active ingredient in “horny goat weed” (HGW) and a traditional Chinese remedy for male sexual function—are an underrecognized source of such compounds. To our knowledge, this is the first reported case of new‐onset psychosis following epimedium use in a person with PD (PwP).

## Case Report

A 59‐year‐old PwP was brought to the emergency department (ED) by family after developing new delusional thinking and disorganized behavior. Family reported 2 days of bizarre conduct, including persecutory delusions, paranoia, and confusion, culminating in a fall with head injury and possible loss of consciousness. He was living independently, without prior cognitive or psychotic symptoms, and was followed regularly by his neurologist.

He was diagnosed with PD 8 years before, presenting with a 2‐year history of left‐hand tremor. There were no overt signs of NPS (including cognitive dysfunction, delusions, or visual hallucinations) upon initial or subsequent evaluations. He was treated with carbidopa/levodopa (CD/LD) 25/100 with partial tremor control but developed motor fluctuations and dyskinesias. Rasagiline and amantadine were trialed but discontinued due to limited benefit. At presentation, his regimen included extended‐release CD/LD (36.5/145 mg, 3 capsules QID) and ropinirole (4 mg QD), with an LEDD of 1124 mg. This dopaminergic regimen had not increased in the preceding 28 months.

In the ED, mental status was notable for paranoid delusions, flat affect, monotone speech, and poor judgment and insight. There was no suicidal or homicidal ideation. He was alert, oriented, and cooperative with fluent, linear speech. On motor examination, he had mild asymmetric parkinsonism without dyskinesias. Noncontrast computed tomography (CT) brain, chest radiograph, and urinalysis were unremarkable. Labs showed mild leukocytosis (15 × 10^3^/μL), hypokalemia (3.2 mmol/L), elevated blood urea nitrogen (BUN) (34 mg/dL), and bilirubin (1.2 mg/dL). Electrocardiogram (EKG) revealed new atrial fibrillation. Urine drug screen was negative for amphetamines, barbiturates, benzodiazepines, cannabinoids, cocaine, opiates, or phencyclidine. Blood alcohol level was <10 mg/dL, and whole blood phosphatidylethanol was <20 ng/mL. Thyroid‐stimulating hormone (TSH), B_12_, folate, and lactic acid were normal.

The patient was admitted for further evaluation. Leukocytosis, BUN, and bilirubin resolved within 48 hours without intervention. Hypokalemia persisted for 10 days and resolved after home hydrochlorothiazide and furosemide were discontinued. Electroencephalogram (EEG) showed mild asymmetry with attenuation of faster frequencies and sleep structures over the right hemisphere, but no epileptiform activity. Lumbar puncture was deferred due to resolving leukocytosis and absence of localizing features (eg, headache, meningismus). Due to concern for hyperdopaminergic tone, ropinirole was decreased from 4 to 2 mg daily. Magnetic resonance imaging (MRI) of the brain was not obtained due to the need for sedation given encephalopathy.

Upon questioning, the patient disclosed taking HGW daily for the past month. He denied taking additional CD/LD or dopamine agonist. He was hospitalized for 3 weeks and experienced hyperactive delirium. He was started on quetiapine and clonazepam, with gradual improvement in delusions and encephalopathy. Post‐discharge, he developed daytime somnolence, prompting dose reductions. One month later, intermittent paranoia and agitation persisted. Pimavenserin was initiated 2 months after discharge, with resolution of NPS noted 3 months later. By 10 months post‐hospitalization, the patient remained asymptomatic neuropsychiatrically while on pimavenserin and a slow taper of quetiapine.

## Discussion

Herbal supplements represent an unregulated market and an important contributor to NPS, including psychosis and mania in susceptible individuals.^4^ In addition to icariin (the erectogenic flavonoid in epimedium), HGW supplements can contain psychoactive compounds like yohimbine and ginseng.^5^, ^6^ Ginseng has been linked to psychosis, although with rapid resolution once offending supplements discontinued.^7^

Although the temporal relationship between HGW use and onset of psychotic symptoms is compelling, concurrent use of dopaminergic medications and mild metabolic abnormalities may have contributed to patient's diathesis to drug‐induced psychotic phenomena. HGW inhibits multiple CYP isoforms, particularly 1A2, which controls ropinirole metabolism.^8^ Ropinirole, as a D2/D3 agonist, may contribute to the development of NPS and PDP.^1^ HGW use may have resulted in higher ropinirole levels through CYP inhibition and lowered the threshold for impulse control disorder (ie, increased sexual behaviors). HGW constituents may have also directly contributed to a hyperdopaminergic state, although studies on neuronal effects of HGW are largely preclinical.^6^, ^9^

Alternatively, or additionally, HGW may have contributed to toxic‐metabolic encephalopathy via acute behavioral change (eg, acute renal injury due to poor oral intake) or cardiovascular effects (eg, cardiac dysrhythmia) from contaminants in HGW (such as yohimbine), or both. The supplement may have contained unlisted psychoactive compounds contributing to NPS. The exact composition of the supplement in this case is unknown, which is a limitation of this report.

Details of the patient's cognitive and psychiatric baseline were limited by the absence of collateral history or formal neuropsychiatric testing prior to presentation. Though symptoms improved after the discontinuation of HGW, paranoid delusions persisted for several months—longer than in previously reported supplement‐induced psychoses in individuals without neurodegenerative disease.^7^, ^10^ As the patient improved with antipsychotics, this case may reflect a potential for sustained or more severe NPS in PwP after HGW supplements.

This case highlights the importance of inquiry about over‐the‐counter and alternative supplements in routine care of PwP, especially when evaluating new‐onset psychotic phenomena or behavioral changes. Products like HGW can exacerbate or unmask neuropsychiatric vulnerability through pharmacologic or interactive effects, reinforcing the need for comprehensive medication and supplement histories.

## Author Roles

(1) Research project: A. Conception, B. Organization, C. Execution; (2) Statistical analysis: A. Design, B. Execution, C. Review and critique; (3) Manuscript preparation: A. Writing of the first draft, B. Review and critique.

J.D.: 1A, 1B, 1C; 3A, 3B

K.M.: 1A, 1C; 3A, 3B

D.L.: 1A, 3B

D.B.: 1A, 3B

## Disclosures

**Ethical Compliance Statement:** We confirm that we have read the journal's position on issues involved in ethical publication and affirm that this work is consistent with those guidelines. IRB approval was not applicable for this brief single case report. Informed patient consent was not necessary for this work. Personal identifiable information was omitted to ensure individual anonymity. No images or videos were submitted to further support anonymity.

**Funding Sources and Conflict of Interest:** No specific funding was received for this work. The authors declare that there are no conflicts of interest relevant to this work.

**Financial Disclosures for the Previous 12 Months:** The authors declare that there are no additional disclosures to report.

## Data Availability

Data sharing not applicable to this article as no datasets were generated or analysed during the current study.
